# Supplementary material for: The Effects of Cannabidiol and Prognostic Role of TRPV2 in Human Endometrial Cancer
Source: Int J Mol Sci. 2020 Jul 29;21(15):5409. doi: 10.3390/ijms21155409 (PMC7432565; doi:10.3390/ijms21155409)
Supplement: Supplementary file 1 [file ijms-21-05409-s001.pdf]

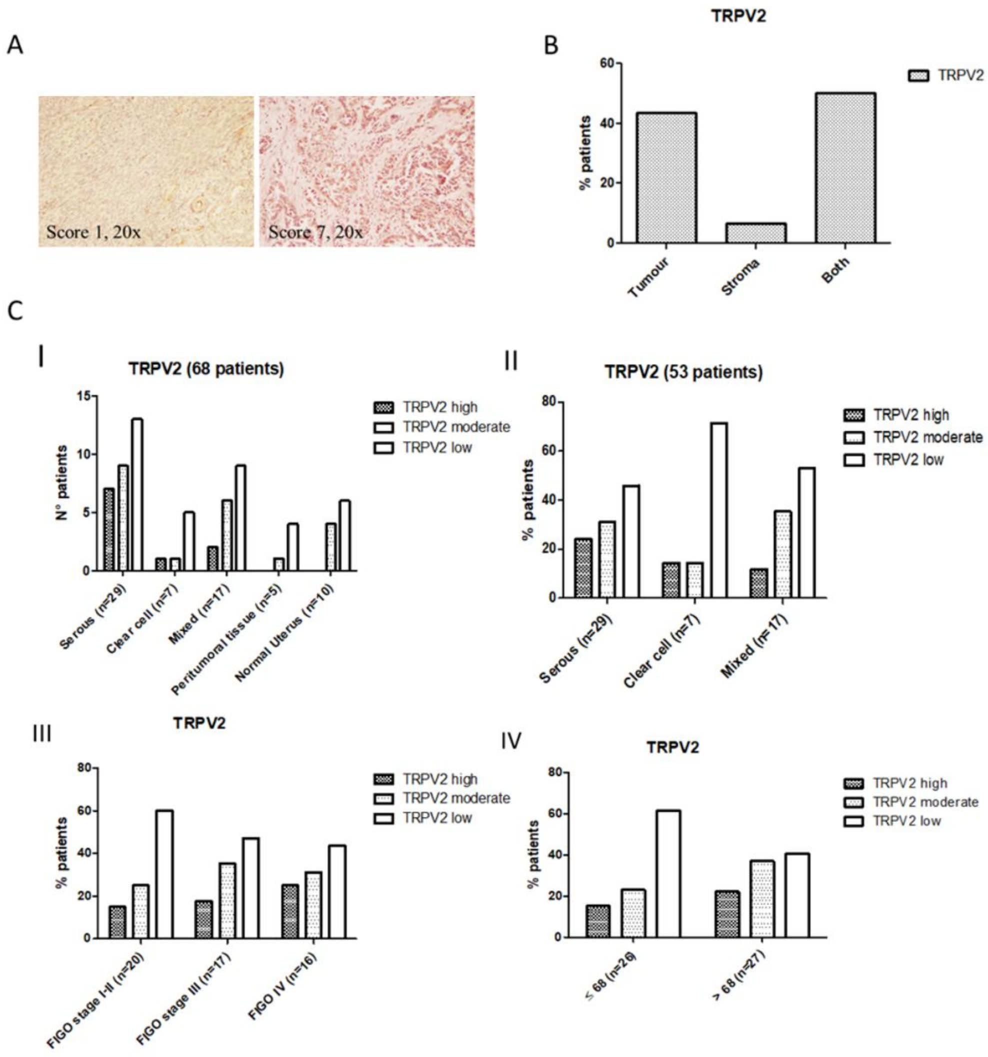

Figure S1. TRPV2 expression in human EC biopsies. (**A**) Representative images of IHC staining and score for TRPV2 in human EC biopsies. Pictures were taken at ×20 magnification (scale bar, 50 μm) (**B**) Percentages of patients classified according TRPV2 distribution inside tumoral mass. (**C**) Classification of patients according to TRPV2 expression and clinicopathological characteristics. I) Number of patients classified according to TRPV2 expression in EC, peritumoral tissue and normal endometrium. II) Percentages of patients classified according to TRPV2 expression in EC. III) Percentage of patients classified according to TRPV2 expression and FIGO stage. IV) Percentages of patients classified according to TRPV2 expression and age

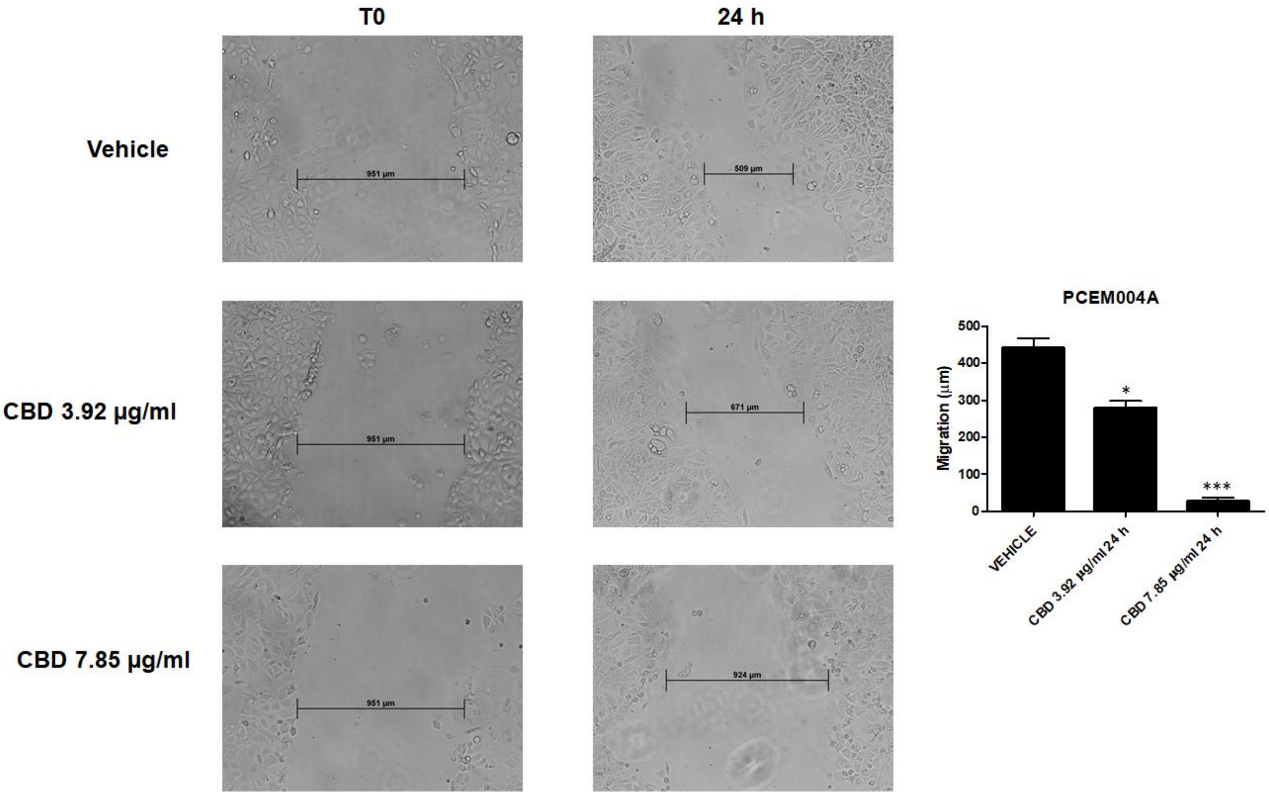

Figure S2. CBD treatment inhibited migration of PCEM004a cells. Wound-healing assays for PCEM004a cells after treatment with CBD 7.85 μg/mL and 3.92 μg/mL for 24 h. All experiments were repeated three times and images were taken at 0 and 24 h (20×). Data are presented as the mean ± SD. * $p < 0.05$ vs. untreated.
